# Cataphoresis

**Published:** 1896-11

**Authors:** Henry W. Gillett


### CATAPHORESIS.[1]

BY HENRY W. GILLETT, D.M.D.

Mr. President and Gentlemen,—It was with some hesitation that I accepted the invitation of your committee to speak to you on this subject this evening, for the reason that I have but little new material to add to what I have already published. Consequently I

---

[1] Read before the American Academy of Dental Science, April 1, 1896.

must confine myself largely to further elaboration of matter already published, and to the consideration of the statements recently published by other workers along this same line.

As an introduction and a probable help to some of you in grasping more fully what follows, let us define certain technical terms which it will be necessary to use. To many of you this will undoubtedly be superfluous, but I find a majority of the profession are not sufficiently familiar with these terms to use them understandingly. I trust, however, that it will not be long before our schools shall recognize the growing demand and the necessity for providing instruction in electricity instead of leaving it to private post-graduate enterprise and the commercial agents of supply-houses, the present most readily accessible sources of instruction for the professional man.

It is well understood by all, I presume, that the term electrical current is used for convenience, and as the result of long habit. In the early history of the development of our knowledge of electricity, it was thought that the manifestations indicated a flowing current comparable to a current of fluid, so we have the term electric current, which seems unlikely to be supplanted. In speaking of this current we need units of measurement of its different qualities. We have these in the ampère, volt, ohm, coulomb, etc.

This term, coulomb, at present one of the least used but most important terms, is the unit of quantity. I refer to it here for two reasons: first, as an aid to the understanding of the ampère, and, secondly, because I believe it will eventually be one of the most commonly used terms. If your bill from your electrical supply company should charge you at a certain sum per thousand coulombs of electricity, it would correspond exactly to the usage of your gas company in their charging a certain sum per thousand cubic feet of gas.

Having a unit of quantity, we next need a unit of current strength or rate of flow. We have this in the ampère, which is the current strength, which allows one coulomb of current a second to pass a given point in the circuit, and corresponds in usage to cubic feet per second of gas or water flow.

For instance, five ampères of current flowing one second deliver five coulombs of electricity, in ten seconds it delivers fifty coulombs.

In medicine we use comparatively weak currents, and consequently measure it in thousandths of an ampère,—milliampères.

In the volt we have the unit of electro-motive force, corresponding to our usage of pounds pressure per square inch in gas, steam, or water.

When we modify the one-hundred-and-ten-volt current so as to use a ten- or twenty-volt current from it, we do something which we may illustrate by the dam of the millwright. Let us suppose he has a twenty-foot dam, and a flume from the bottom of it for his main millwheel, where he may use all the pressure he can get from his dam; but for some other small machinery, let us suppose, he leads a flume out five feet from the top to run a small water-wheel requiring less pressure. With this selector we tap the one-hundred-and-ten-volt circuit, with an adjustable contact, and draw off from it any part of forty volts in pressure that we may wish to use.

One other term, resistance, has an obvious meaning, it is the opposite of conductivity. You know, for instance, that copper conducts electricity well, iron conducts it less readily, and German silver least readily of our commonly used metals; conversely, we say that the resistance of copper is less than that of iron, and that of German silver greater than that of iron; that the resistance of distilled water is very high; that of a sodium chloride solution low; that of the tissues of the body higher than the substances we have mentioned except pure water, and that of a dry tooth very high. The resistance of a conductor also depends upon its dimensions. A large wire will conduct more current than a small one; a large surface of the body covered with an electrode will conduct more current without pain than will a less portion of the same surface. We measure this resistance in ohms.

The ohm is the resistance of a column of mercury of a certain size and length; roughly, it corresponds to the resistance of four hundred and sixty feet of ordinary telegraph wire.

The terms positive and negative scarcely need defining. The positive, indicated by the plus (+) sign, being applied to the current coming from the dynamo or battery; the negative, indicated by the minus (—) sign, being applied to the return current. Electrode is a term commonly used to describe the applicators used in administering electricity. The positive electrode is often called the anode, and the negative the cathode. We have several subdivisions of electricity and kinds of electric current, but we are concerned to-night with but one kind, the continuous or galvanic current, such as is used for incandescent lighting, or is obtained from any form of primary battery.

Now, it was discovered years ago (1858, to be exact) that the galvanic current would carry with it into the tissues of the human body such liquids as tincture of aconite and chloroform.

Richardson, who made this discovery, subsequently retracted his statements, but others took the matter up at different times. Late in the eighties Peterson, Morton, and others did valuable work in developing the principle and process, which had come to be known as cataphoresis. Many other terms have been used to describe it, but this seems still to be the favorite. In a paper published in the February number of the INTERNATIONAL DENTAL JOURNAL, I quoted many of Dr. Peterson's experiments and his references to the earlier history of the principle. In that paper I attempted to give, as concisely as possible, an outline of the development of the use of the principle in medicine.

Cataphoresis has been for some years a process of recognized value to the electro-therapeutist. For further information on the history of its use in medicine I would refer you to the article mentioned and to the references made there.

Now let us consider what happens when we perform cataphoresis in any tissue.

We apply a suitable galvanic current to the tissue by means of suitable applicators or electrodes, the current flows through the tissue from the positive to the negative electrode, and it carries with it a portion of any fluid having the right properties, such fluid being placed on or in the positive electrode.

This property of the galvanic current seems to be partly or largely a mechanical effect. It is stated by Morton that he has driven powdered graphite deeply into the sweat-follicles of the skin by this means. A globule of mercury can be driven back and forth in a tube containing dilute sulphuric acid by alternating the application of the positive and the negative current to wires dipping into the fluid at each end.

I have here an example of what happens if you use a copper electrode. In this case we have decomposition of the copper electrode itself, then cataphoresis of the resulting copper salt. It was done in this way. The copper wire was thrust into this piece of steak and made the positive electrode, another wire was thrust in at another point and the current turned on. You observe the spreading and penetration of the green stain into the meat.

With your permission I will now quote from a previous article of mine some experiments bearing more directly on our use of the current. (See February number of the *Dental Cosmos.*)

You will find at the end of the same article several cases from practice, and others are cited in my article in the February *Dental Cosmos.* I might quote to you many more, but it seems unnecessary.

Now let me define what I meant a few moments ago by a suitable galvanic current. The current from any set of primary batteries or any continuous current lighting system answers the requirements. It is, however, very desirable that the current supply be steady. I am told that your system here in Boston is not satisfactory in that respect. This fluctuation, of course, may come either from unsatisfactory conditions at the central station, or from large intermittent demands upon the system made by the running of machinery subject to sudden stopping and starting. When a suitable street current is not at command, a dry battery outfit seems to me to offer the best source of current-supply for this purpose. In fact, I am beginning to question whether the dry battery outfit, which the manufacturers of the selector have prepared to go with it, is not the best source of current-supply. It certainly has the merit of steadiness of current and of not needing attention in caring for it.

Now as to electrodes: for the negative, the ordinary sponge-covered electrode is suitable. The sponge, however, soon becomes dirty, and I prefer to strip it off and use for each patient a fresh piece of cottonoid or other absorbent material. The positive electrode varies according to the part to which it is to be applied. For obtunding or bleaching dentine a platinum point is needed, which is to be applied to cotton placed in the cavity or to have cotton wound upon it. For obtunding soft tissues the most successful electrode is that suggested by Morton, having a reservoir which feeds the solution to be used to the absorbent layer between the metal of the electrode and the skin. It is necessary for satisfactory results from cataphoresis that the metallic conducting medium shall come close to the tissue to be effected. As to drugs, there are many that may be used, and more will undoubtedly be found useful for different purposes, but to-night I wish to confine myself especially to three. The first is cocaine, with which nearly all my own work has been done. When an aqueous solution of cocaine of fifteen to thirty per cent. is placed in a sensitive cavity so insulated from the soft tissues that the current must go through the tooth, and the positive electrode is applied to this cotton, and a current of suitable strength is passed for a varying length of time, enough of the cocaine will be carried into the dentine to induce there the normal cocaine effect. The time required to produce this effect varies with the conditions.

For absolute insensibility to such cutting as we need to do in preparing a cavity for filling, ten to fifteen minutes is average time. In some cases the time will be less than this, and in others it may require considerably more. With patients very sensitive to the electric current, it takes more time to reach a voltage which will accomplish the work quickly. A voltage of ten or twelve is sufficient, fifteen or twenty is more rapid in its effects, and thirty or more may be used in some cases. A better method of measurement, and in fact the only correct one, is to use a milliampère meter and gauge your dosage by that. The same voltage produces very different effects as regards quantity of current in different teeth. If we wish to bleach a pulpless tooth we cleanse the cavity, and fill the upper third of the canal as usual, and apply on cotton to the exposed dentine a twenty-five-per-cent. aqueous solution of pyrozone. This is made by shaking two parts of twenty-five per cent. ethereal pyrozone with one part of water, and allowing the ether to evaporate. The tooth being insulated by the rubber, such current as the patient can comfortably bear is pressed, and the tooth will be bleached in a comparatively short time, provided, of course, the coloring is one to be effected by the pyrozone.

Guaiacol has been advanced as a candidate for use as an anæsthetic agent. Some time ago it was announced in Paris that guaiacol had marked anæsthetic properties, and that it was superior to cocaine in this respect. It was recommended to be used hypodermically, but with the warning that it would produce sloughing at the point of injection. Acting upon this hint Morton claimed to have found that guaiacol used cataphorically on the skin would produce anæsthesia in about half the time that was required for cocaine solutions to produce the same effect. He tried the addition of cocaine to guaiacol, and used it on two teeth, on mucous membranes of the mouth for implantation, and for other operations. He reported excellent results, but it has since been stated that there is considerable sloughing when it is used cataphorically on mucous membranes. I have not yet ventured to try it in sensitive dentine, though I have seen it successfully used. I fear its escharotic properties may do injury to the tooth. McKesson & Robbins, who have made up the combination of guaiacol and cocaine which they call guaiacocaine, assure me through their agent, that we may hope to see a guaiacol free from these escharotic properties. Guaiacol, as you know, is a synthetic creosote. It is stated that it contains one and a half per cent. of hydroxyl, to which are ascribed its escharotic properties. It is hoped by the firm named that they may be able to get a product free from hydroxyl. If they are able to realize this hope, and the results are what they expect, we shall undoubtedly have a very valuable drug in the combination guaia-cocaine. Its action will be more prompt than that of cocaine alone, and the guaiacol will serve a very valuable purpose when the combination is used in soft tissues in retaining the cocaine, and preventing its rapid distribution to the system. It is stated that when cocaine is added to a mixture of guaiacol and water, the guaiacol takes up eighty-six per cent. of the cocaine, leaving only fourteen per cent to the water. We now come to the apparatus required and the technique of making the application to sensitive dentine, or for bleaching, the only difference being that for bleaching you may use more current, and may add the current in larger steps. A switch is provided on the one-hundred-and-ten-volt selector for this use.

The ordinary electric apparatus used by the medical profession is very nearly useless in making applications to sensitive dentine. The living tooth is much more sensitive to the irritation of the electric current than most other tissues of the human body. Some subjects are so sensitive to its effects that the increase of pressure even in the quarter-volt steps given by this selector is apparent to them, in the early stages of the application to sensitive dentine.

On the other hand, some subjects are able to bear the full one-hundred-and-ten-volt current when it is choked down in flow, as described by one of your Boston practitioners in the March INTERNATIONAL DENTAL JOURNAL. I have taken such a current as he describes through sensitive dentine without finding it unbearable, but for the subject markedly sensitive to the galvanic current such a remedy is worse than the disease. I began my work a year ago with an apparatus giving just about the results he describes, but soon found it necessary to modify it, and when I found the key to the problem I also found my original expensive apparatus to be useless. I would say that the dangers referred to in the article mentioned are imaginary in a properly-made apparatus.

This fractional volt-selector, made for me by the Electro-Therapeutic Company, of New York, has been especially adapted to the needs of the dental operator. As I have intimated, it is so arranged that the current is added in quarter-volt steps. The ability to do this is an essential factor to the general use of this method of obtunding sensitive dentine. Given this ability to control the current, and any sensitive cavity that is so situated that the current can be made to travel through it, and through the tooth, and anæsthetics can be forced into the dentine with no more discomfort than will be readily borne by sensitive patients. The dial at the top of the selector is a volt index. It is not absolutely, but is very nearly, correct for such currents as are likely to be used with it. The electrodes needed are the ordinary sponge electrodes, and a needle-holder for the piece of iridio-platinum wire for the positive electrode. A milliampère meter is very desirable also. With this selector it cannot be said to be an absolute essential, since you have in the voltage and in the patient's sensations two guides. It is, however, impossible to know what quantity of current you are using unless you do have a meter, and I would not willingly dispense with one. It should be so arranged as to register five milliampères on a scale a couple of inches long, so as to have a considerable movement of the needle for each milliampère.

The technique of administration is simple. If possible, apply the rubber dam. If the cheeks and mucous membranes can be kept away, and the parts kept dry enough so that the current will not flow off through the other tissues, it will do as well, but it is usually much easier to put on the rubber at once. Next see that any metal fillings, near enough so that the current may reach them either by contact with the cotton, the electrode itself, or by the surplus of the anæsthetic solution flowing along to them, be insulated by covering with soft gutta-percha, wax, varnish, or other non-conductor. Select a pellet of cotton loosely rolled, and of such size and shape as will about fill the cavity, saturate it with a freshly made twenty- or twenty-five-per-cent. aqueous solution of cocaine, and place it in the cavity. The sponge of the negative electrode should be well wet with water or dilute salt solution. The patient may hold it in the hand if preferred. I generally let either the patient or my office attendant hold it on the side of the face or neck. Having the selector conveniently placed at the back so it can be reached with the left hand, the current turned on at the switches, and the knob turned back till the needle stands at zero, apply the positive electrode to the cotton, and slowly turn on the current by the knob on top. Keep your contacts steady and watch your patient. Usually at from three to six volts the patient will begin to feel the sensation due to the flow of the current. This first sensation cannot be called pain: most subjects speak of it as a feeling of cold. As the patient begins to show that the sensation is beginning to be painful, pause in the turning-on process, or even turn it back a bit. Explain that after a moment's rest the sensation will grow less. Take this opportunity to explain that the current is entirely under control; that it is not necessary to give pain, but the current should be added as fast as may be borne without real discomfort, and that the stronger current works much faster. After a period varying from fifteen seconds to two minutes you will usually be able to go on, adding current gradually. If I am able to get as high as a fourteen- or fifteen-volt current in the first seven or eight minutes, I generally continue the application only nine or ten minutes. If it takes me longer to get to fifteen volts, I am apt to continue the application for fifteen minutes, especially if I have a very sensitive cavity to deal with. I have absolutely benumbed the dentine of cavities in seven minutes for patients who could take a good deal of current. You soon get to know about what each patient needs. If, in turning on current, you find that after ten volts or so have been turned on you can add two or three volts at a time, you may know that the cocaine effect is well under way. When the dentine is anæsthetized you can usually go on adding current rapidly up to thirty or forty volts if you wish. I consider a voltage of fifteen or twenty an average application. If you have a milliampère meter, and find it recording anything from one-third of a milliampère to two milliampères, you may be sure of ultimate success, even if you can use only a low voltage. Very marked sensitiveness to the low voltages is present when there is an exposed pulp below the softened dentine. An exposed pulp will not readily bear as high voltages as dentine will, and in these cases your meter will record more current. Having completed my application, I turn the current back nearly to zero, and remove the negative electrode first. If the patient is one not very sensitive to the current I do not trouble to turn back to zero, but break contact gradually by sliding the negative electrode off slowly. I then go on with the excavation as usual, bearing in mind that I may penetrate through the anæsthetized layer. If this is done a second application may be made, in which you will be able to turn on current much more rapidly, and need shorter application. In my sensitive cavities I find I make up the time by reason of the increased speed possible in doing the cutting afterwards. In many cases I have been able to anæsthetize, completely and painlessly, dentine for patients for whom I have previously been unable to find any relief. I find no cases of sensitive dentine that cannot be controlled when cocaine cataphoresis can be employed. I anticipate being able to control them with less expenditure of time as we gain experience with other drugs.

